# Evaluating Nursing and Midwifery Students’ Self-Assessment of Clinical Skills Following a Flipped Classroom Intervention with Innovative Digital Technologies in Bulgaria

**DOI:** 10.3390/nursrep15080285

**Published:** 2025-08-06

**Authors:** Galya Georgieva-Tsaneva, Ivanichka Serbezova, Milka Serbezova-Velikova

**Affiliations:** 1 Institute of Robotics, Bulgarian Academy of Sciences, 1113 Sofia, Bulgaria; 2 Faculty of Public Health and Healthcare, University of Ruse “Angel Kanchev”, 7017 Ruse, Bulgaria; iserbezova@uni-ruse.bg (I.S.); milka.serbezova@googlemail.com (M.S.-V.)

**Keywords:** flipped classroom, healthcare, nursing education, video algorithms, clinical simulation, clinical problem-solving, virtual reality, self-assessment, digital competence, injection techniques, blended learning

## Abstract

**Background/Objectives**: The transformation of nursing and midwifery education through digital technologies has gained momentum worldwide, with algorithm-based video instruction and virtual reality (VR) emerging as promising tools for improving clinical learning. This quasi-experimental study explores the impact of an enhanced flipped classroom model on Bulgarian nursing and midwifery students’ self-perceived competence. **Methods**: A total of 228 participants were divided into a control group receiving traditional instruction (lectures and simulations with manikins) and an experimental group engaged in a digitally enhanced preparatory phase. The latter included pre-class video algorithms, VR, and clinical problem-solving tasks for learning and improving nursing skills. A 25-item self-report questionnaire was administered before and after the intervention to measure perceived competence in injection techniques, hygiene care, midwifery skills, and digital readiness. **Results**: Statistical analysis using Welch’s t-test revealed significant improvements in the experimental group in all domains (*p* < 0.001). Qualitative data from focus group interviews further confirmed increased student engagement, motivation, and receptiveness to digital learning tools. **Conclusions**: The findings highlight the pedagogical value of integrating structured video learning, VR components, and case-based learning within flipped classrooms. The study advocates for the wider adoption of blended learning models to foster clinical confidence and digital competence in healthcare education. The results of the study may be useful for curriculum developers aiming to improve clinical readiness through technology-enhanced learning.

## 1. Introduction

Nursing is a highly dynamic profession that requires professional resilience and the ability to function effectively under emotional and intellectual stress. Nursing is an integral part of healthcare that aims at supporting individuals, families, and communities in achieving, maintaining, and restoring optimal health and quality of life. At its core, nursing is grounded in a holistic approach to patient care, encompassing activities in health promotion, prevention, treatment, rehabilitation, and palliative care, applied in the context of individual needs and the characteristics of different age groups. In modern practice, nursing education should not only prepare students with theoretical knowledge, but also with practical skills, critical thinking, and technological literacy in order to be able to effectively respond to the challenges of the dynamically changing healthcare environment.

In recent years, the integration of digital technologies in healthcare education has advanced rapidly, transforming the way students acquire, practice, and assess their clinical competencies. Self-assessment enables nursing students to critically reflect on their skills, identify gaps, and engage more actively in the learning process. It is emerging as a key educational tool that allows students to evaluate their knowledge, skills, and readiness for real-world clinical practice. Self-assessment fosters metacognitive awareness, promotes autonomy, and serves as a reliable predictor of future performance when combined with formative and summative evaluations.

However, when comparing self-assessment to mentor evaluations, Manero-Solanas et al. [1] reported notable discrepancies, despite significant post-intervention improvements. These differences, often attributed to overestimation or overconfidence, underline the importance of triangulating self-assessment with external evaluation to ensure accuracy. Again, the results show statistically significant differences in key indicators, with self-criticism remaining strongly present. In a medical education context, one study [2] highlighted that self-perceived communication skills following the delivery of bad news did not consistently align with instructor ratings, reinforcing the need for structured and validated assessment frameworks.

Protocol-based studies and experimental studies have demonstrated that virtual reality (VR) simulations can significantly enhance nursing students’ clinical confidence and critical judgment. The authors of reference [3] reported increased confidence, task engagement, and self-assurance when using VR-based home care scenarios compared to control groups exposed to video-based materials. A pre- and post-intervention questionnaire with a Likert scale was employed to assess students’ confidence in performing tasks during home visits.

A focus group study [4] revealed that nursing students actively seek immediate, difficulty-adjusted feedback and interactive case scenarios—factors that are closely associated with the accuracy and effectiveness of self-assessment. The findings highlighted students’ preference for VR solutions that directly support their self-assessment and learning processes. In this case, self-assessment was operationalized through subjective responses in which students described their learning needs and perceived preparedness during the VR training, constituting a non-formalized, thematically coded form of self-evaluation. A meta-analysis [5] identified effect sizes ranging from 0.2 to 0.9 in clinical competence following VR-based interventions, demonstrating consistent benefits across both perceived and objectively measured skills.

Integrating innovative technologies such as VR, serious games, and flipped classroom models has the potential to further enhance nursing students’ capacity for accurate self-assessment by offering interactive tools that reinforce procedural knowledge, encourage reflective practice, and personalize feedback. Research by Padilha et al. [6] and Koivisto et al. [7] has shown that VR-enhanced simulation improves procedural accuracy, clinical judgment, and student confidence in nursing practice. Augmented Reality (AR) is also gaining momentum, particularly in surgical education.

In Bulgaria, the educational pathway for nursing students follows a structured and accredited system. The Bachelor of Science in Nursing and Midwifery is a four-year university program (240 ECTS), which includes both theoretical instruction and clinical practice. Clinical education is integrated throughout the curriculum and is conducted in healthcare institutions under the supervision of qualified mentors. Traditionally, practical training takes place in hospitals and outpatient settings, focusing on real patient care. In recent years, and particularly following the COVID-19 pandemic, digital learning tools, simulation-based education, and online platforms have been increasingly adopted. The University of Ruse supports blended learning through its dedicated e-learning platform [https://e-learning.uni-ruse.bg/], which facilitates flipped classroom models, video-based instruction, and virtual simulation scenarios. This integration enables students to develop clinical, procedural, and communication skills in a digitally enriched environment.

An interactive educational system has been created at the Angel Kanchev University of Ruse [8] for nursing and midwifery that incorporates educational videos, serious games, and problem-based scenarios. The system incorporates feedback from both students and faculty and focuses on developing innovative tools [9,10] for clinical skill acquisition, particularly in procedures like injections.

In a related study, Zlatarov et al. [11] developed a web-based platform designed to track the personalized learning paths of doctoral students, illustrating the broader applicability of digital systems for individualized progress monitoring and self-regulated learning. The flipped classroom (FC) model has emerged as a transformative pedagogical approach in nursing and healthcare education, aimed at promoting active learning and deeper engagement. Evidence suggests that the FC model enhances critical thinking, satisfaction, and academic performance among nursing students.

A meta-analysis by Park et al. [12] showed significant improvements in clinical competence, critical thinking, and student satisfaction, highlighting the impact of flipped learning on the development of key professional skills in nursing. The authors of [13,14] confirm that the flipped classroom significantly improves learning outcomes and student engagement in the health sciences when combined with active methods and technological support. Scientific research shows that video training significantly improves motor learning and practical skills by activating visual–motor associations and supporting imitation and retention processes [15,16].

Several studies have highlighted the pedagogical value of simulation-based learning, even in contexts with limited access to modern technology. However, the results suggest that the effectiveness of such interventions can be further enhanced by the following:

- Integrating flipped classroom approaches [17];
- Formalizing structured discussion sessions [18];
- Incorporating communication and teamwork scenarios [19];
- Expanding VR components from demonstration to fully interactive applications [20].

Implementing these enhancements would support the development of both cognitive and practical skills among nursing and midwifery students, ultimately contributing to more effective clinical learning and professional readiness.

Empirical studies conducted internationally highlight a range of positive effects as-sociated with the use of virtual reality in healthcare education, including the following:

- Improved knowledge acquisition [21];
- Increased self-confidence and clinical precision [22];
- A safe environment to practice and learn from errors without real-world consequences;
- Enhanced student motivation and engagement [23];
- Opportunities for standardized and repeatable simulations [24].

At the same time, several challenges to implementing VR in education persist, including the following:

- High initial investment in equipment and infrastructure;
- The need for faculty training and development;
- The risk of technology overload without meaningful pedagogical value.

Aim of the Study: This study explores nursing and midwifery students in Bulgaria’s perceptions of digital transformation and the flipped classroom methodology, presenting a flipped classroom educational framework that is suitable for nurses learning basic nursing technologies. Specifically, the aim is to assess their self-reported clinical competence, motivation for digital learning, and openness to using digital technologies, before and after a structured training intervention. The findings aim to inform future teaching strategies and support the evidence-based integration of innovative tools into health education, with a focus on feasibility, perceived value, and implementation challenges.

## 2. Materials and Methods

### 2.1. Educational Design and Methodological Aspects

The intervention model in this study is built on the basis of modern pedagogical paradigms that emphasize active and independent learning, technology-mediated learning, and simulation-based training. The educational design of the experimental group is based on an extended flipped classroom model, in which theoretical content is mastered at home and classroom time is reserved for practical application [25,26].

The practical phase in both groups included training using low-to-mid-fidelity manikins, allowing for the safe practice of skills in a controlled environment. As shown in previous studies [27,28], even low-fidelity simulations offer measurable benefits for acquiring clinical competencies.

The theoretical foundations of the study draw upon established educational frameworks—Vygotsky’s social constructivism [28], Mayer’s cognitive theory of multimedia learning [29], Kolb’s experiential learning theory [30], and the INACSL Standards of Best Practice in Simulation [27]—each of which directly informs the development and assessment of specific competencies measured by the questionnaire.

Kolb’s experiential learning theory provides the pedagogical basis for structuring the learning cycle, and is divided into the following categories: concrete experience (simulations and clinical cases—Q1–Q19), reflective observation (real-time feedback and video analysis—Q20–Q22), abstract conceptualization (self-assessment and digital content—Q23–Q25), and active experimentation (repeated practice and skills transfer—Q1–Q10). The progression through this cycle supports the development of both procedural and reflective competencies.

Vygotsky’s sociocultural theory, particularly the concept of the zone of proximal development (ZPD), underlies the importance of facilitated learning and scaffolding during simulation and practice. Questions related to teamwork, communication, and instructor-led sessions (Q20–Q22) are directly linked to collaborative learning processes that enable students to operate within their ZPD, gradually becoming more autonomous.

Mayer’s cognitive theory of multimedia learning justifies the use of videos, digital modules, and VR in the flipped classroom model. This aligns with Q23–Q25, which assess students’ motivation and readiness for digital learning, and supports retention and transfer of procedural knowledge, as measured in Q1–Q19.

The INACSL Standards of Best Practice serve as the clinical simulation framework, emphasizing pre-briefing, scenario fidelity, debriefing, and outcome-based assessment, which are directly tied to students’ perceived competence in performing technical procedures (Q1–Q10), communication and ethics (Q20–Q22), and self-efficacy in simulation-enhanced learning (Q23–Q25).

The theoretical framework of this study is grounded in four established approaches—Vygotsky’s sociocultural theory, Kolb’s experiential learning cycle, Mayer’s cognitive theory of multimedia learning, and the INACSL Standards of Best Practice in Simulation. Each framework contributes distinct principles related to social interaction, learning progression, digital engagement, and simulation authenticity in clinical education. Based on this interdisciplinary foundation, the questionnaire was designed to enable valid and theoretically informed assessment of key clinical and cognitive competencies.

Previous clinical studies [1,6,31,32,33] on similar topics were also taken into account.

**Figure 1:**
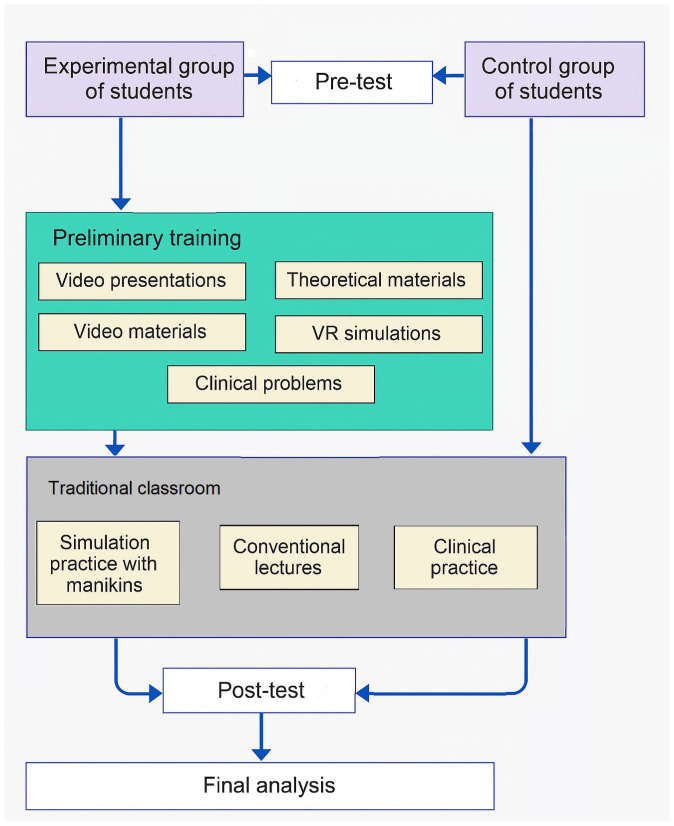
Organization of the research conducted.

Figure 1 shows a diagram of the organization of the training provided.

#### 2.1.1. Flipped Classroom Includes

1. Educational video materials.

The flipped classroom component was implemented using the official e-learning platform of the University of Ruse (https://e-learning.uni-ruse.bg/), based on Moodle. Platform analytics (activity tracking and time logs) were used to ensure student engagement.

The University of Ruse e-learning platform (https://e-learning.uni-ruse.bg/) was used as the central environment for hosting educational videos, digital presentations, and theoretical resources. This platform fully aligns with the national academic standards and curriculum requirements for nursing and midwifery education. The materials uploaded to the platform correspond to the competencies defined in the Bulgarian educational standards and reflect the structure of the national nursing training modules.

Lecturers from the Department of Healthcare at the “Angel Kanchev” University of Ruse developed instructional videos hosted on YouTube. Designed to support autonomous learning and enhance the acquisition of injection techniques, these materials provide structured, step-by-step guidance for mastering essential nursing procedures. The methodological framework aligns with established principles of healthcare pedagogy, ensuring clarity, consistency, and practical skill development. In a flipped classroom, students used nine videos (each lasting 4 to 14 min, approx. 60 min total duration) created by the University of Ruse professors and one created by the Medical University of Plovdiv. The materials were reviewed 3–5 days before the respective practical session, and demonstrate the following procedures:

✓ Intramuscular injection (https://www.youtube.com/watch?v=r20PlDVXsHo);
✓ Intradermal injection (https://www.youtube.com/watch?v=-FZfBHntg1Y);
✓ Subcutaneous injection (https://www.youtube.com/watch?v=a4QTSo9opdQ);
✓ Intravenous injection (https://www.youtube.com/watch?v=xF5GWEfxq0g);
✓ Intravenous infusion (https://www.youtube.com/watch?v=lAP2IVV1hOI).

**Figure 2:**
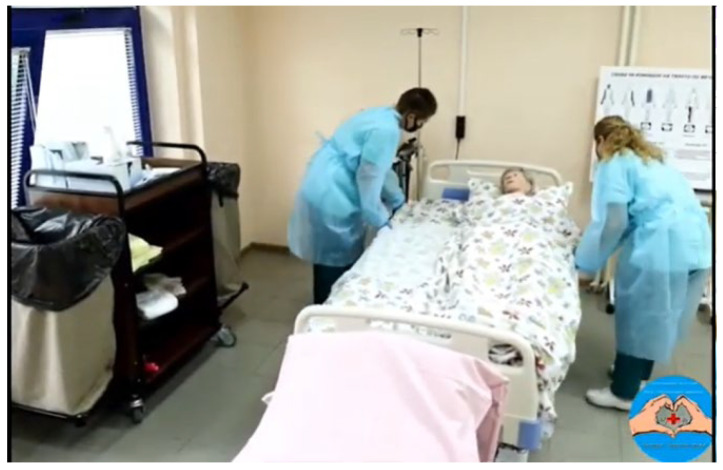
Hygienic toilet using the “dry bathing” method; changing underwear for a seriously ill person (https://www.youtube.com/watch?v=7iv7Wg8oJAo).

**Figure 3:**
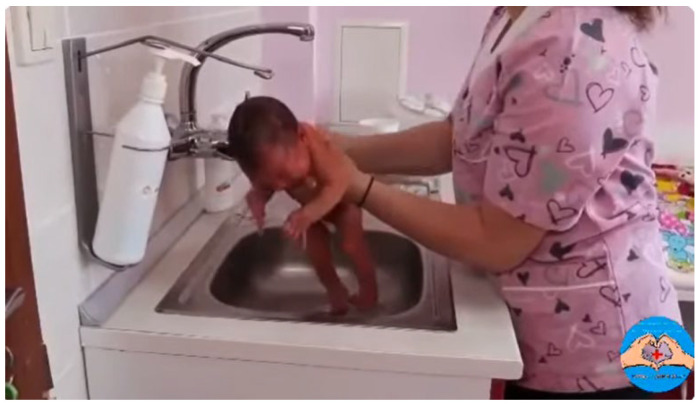
Bathing a newborn (https://www.youtube.com/watch?v=ArA9BNZF0w0).

**Figure 4:**
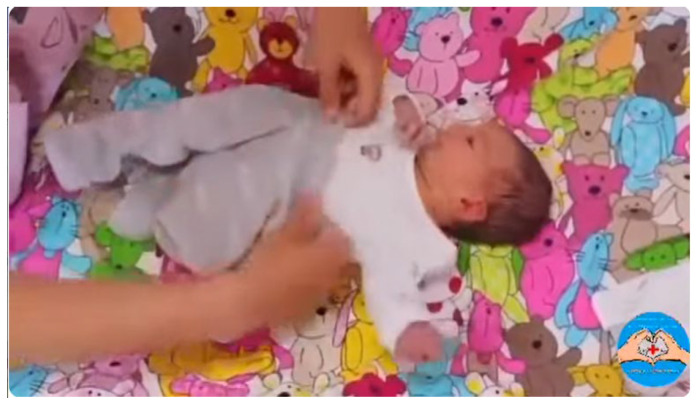
Dressing a newborn (https://www.youtube.com/watch?v=ArA9BNZF0w0).

Videos covering basic medical care activities, such as personal hygiene of bedridden patients and neonatal care, included the following:

✓ The nature and importance of personal hygiene for patients (https://www.youtube.com/watch?v=rnLNzBJAkxk); dry bathing and changing bed linen for immobile patients (Figure 2, https://www.youtube.com/watch?v=7iv7Wg8oJAo); bathing, swaddling, and dressing a newborn (Figure 3 and Figure 4, https://www.youtube.com/watch?v=ArA9BNZF0w0);
✓ Evidence-based management of the third stage of labor (https://www.youtube.com/watch?v=OM222mgzyvM).

Video material about urethral catheterization, which is freely available, was created by the Medical University—Plovdiv. (https://www.youtube.com/watch?v=vVKt7z1lVz0).

2. Educational VR applications include clinical scenarios for the safe practice of various medical skills. These simulations allowed students to visualize and repeatedly rehearse each procedural step in a safe, immersive environment, thus increasing their confidence and readiness for subsequent practical training with manikins. The following were used:
  - SimX—VR scenarios for injection and intravenous procedures;
  - OMS—VR simulations for venous and urinary catheter placement.
3. Video presentations: 15 video presentations on the topics studied, created by Ruse University lecturers, were available through the university’s electronic platform.
4. Test teaching materials: 10 descriptive algorithms for the sequence of actions in 10 types of manipulations and injection technologies were created by Ruse University lecturers and used by students.
5. Four formative quizzes for self-assessment were created and used.
6. Clinical case studies.

Within the innovative educational framework applied to the experimental group, nursing students engaged in pre-designed clinical case studies designed to enhance critical thinking and clinical judgment in key areas such as injection techniques, hygiene care, and urethral catheterization. Each case reflected a realistic clinical practice scenario: administering a subcutaneous injection to a diabetic patient, performing daily hygiene on a bedridden patient at risk of decubitus ulcers, or performing catheterization on a patient with urinary tract disorders. These cases encouraged autonomous decision-making, risk identification, and the implementation of appropriate medical interventions.

To maximize the learning potential of the simulation experience, all sessions in the experimental group were followed by structured debriefing discussions. These debriefings were designed to consolidate knowledge acquired through asynchronous materials—such as instructional videos, VR simulations, digital presentations, and textual content—and to promote reflective learning. The PEARLS framework (Promoting Excellence and Reflective Learning in Simulation) was adopted, integrating learner self-assessment, focused feedback, and collaborative discussion. This model facilitated critical reflection on clinical decision-making, procedural accuracy, teamwork, and communication. It also helped bridge theoretical knowledge with practical execution, ensuring alignment between digital preparation and hands-on competence.

#### 2.1.2. Traditional Training

1. Traditional lectures—4 weeks of 6 lectures per week.
2. Simulation session, which included hands-on procedural practice using anatomical models (manikins) and selected virtual case scenarios (2 weeks, 4 h per week). Low-to-medium-fidelity manikins were used, allowing for the safe practice of skills in a controlled environment.

The anatomical models utilized during training encompassed the following:

Injections: students practiced intramuscular, subcutaneous, intradermal, and intravenous injections, as well as intravenous infusion techniques. The manikins provided tactile feedback and included visible venous structures to enhance procedural realism.

Hygiene care: full-body manikins were used to simulate bedridden patients, enabling students to perform bed baths, oral hygiene, perineal care, and cleaning of the eyes, nose, and ears using standard aseptic techniques.

Childbirth: a birthing simulator was employed to demonstrate and practice the second stage of labor, including assisted delivery, shoulder dystocia management, and postpartum monitoring.

**Figure 5:**
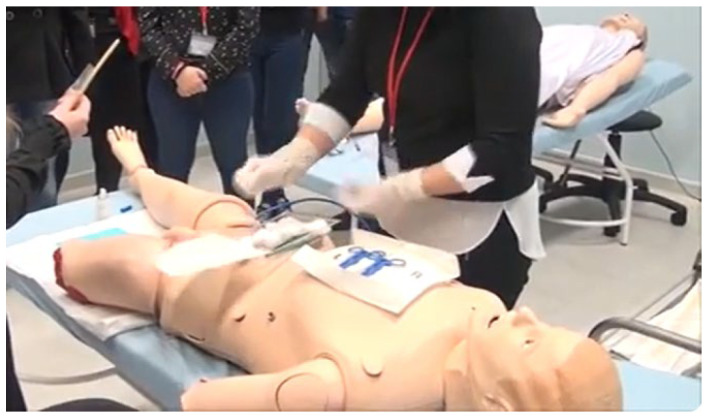
Urethral catheterization. Simulation Center at Medical University of Plovdiv. (https://www.youtube.com/watch?v=vVKt7z1lVz0).

Catheterization: urethral catheterization was practiced using gender-specific manikins that provided realistic anatomical resistance, fluid return, and sterile field setup (Figure 5; instructional video provided by the Simulation Center at the Medical University of Plovdiv).

Clinical scenario training: scenario-based simulations included wound dressing, monitoring of vital signs, ostomy care, and mobility assistance, supporting the integration of clinical reasoning with procedural skills.

All facilitators were university faculty members from the Department of Health. They underwent a two-hour preparatory workshop focused on scenario guidelines, facilitation techniques, and structured performance assessment. Each simulation session followed a standardized structure that included instruction, skill practice, and discussion.

3. Clinical practice (2 weeks of 4 h).

As part of the nursing curriculum at the “Angel Kanchev” University of Ruse, students participate in a structured clinical practice conducted in real hospital settings. The practicum is conducted for two weeks, for one day each week (4 h per day), allowing them to fully immerse themselves in the hospital environment. During this period, students have the opportunity to apply their theoretical knowledge and practical skills in real clinical contexts under the supervision of licensed nurses and clinical instructors. Clinical practice is an essential component of competency development, supporting the transition from simulated to real care and reinforcing key aspects of patient communication, technical procedures, and ethical behavior in professional healthcare settings.

### 2.2. Respondents

The participation of respondents in the surveys was voluntary, completely anonymous, and required prior informed consent for the study. There were total of 230 students in the studied specialty at the university. A total of 228 students (99% voluntarily identified) participated in the surveys, of whom 38 students were randomly selected for the experimental group and the remaining 190 students were the control group. The respondents were from the “Angel Kanchev” University of Ruse, studying the specialties of nursing and midwifery. The participating students were from the second to fourth years. All respondents were women aged 20 to 41 years.

Distribution of respondents:

For 190 CG: 68 second year, 64 third year, and 58 fourth year;

For 38 EG: 14 second year, 12 third year, and 12 fourth year;

Focus group (part of EG): 2 second year, 2 third year, and 2 fourth year.

The study was conducted in April 2025. Students were informed that the purpose of the study was to collect feedback on the perception and effectiveness of digital learning and electronic platforms among healthcare students. The responses received were taken into account to improve the quality of digital learning, as well as to improve e-teaching platforms and methods.

To complement the quantitative results, a focus group interview was conducted with six volunteers from the experimental group after the training intervention. These students were selected through purposive sampling, based on their active participation during the training and their availability for feedback after the intervention. The focus group was made up of two students from each course—second, third, and fourth years (all were women aged 23 to 29). The focus group was moderated by a faculty member involved in the research team, who was trained in qualitative research methods to ensure impartial facilitation and to promote open discussion among participants.

The aim was to gather opinions on their experiences, perceptions, and suggestions for improving the digital and simulation learning process. The focus group provided an opportunity to explore the students’ perspectives in more depth, especially regarding the effectiveness of the flipped classroom components, the clarity and usefulness of the procedural video algorithms, and their impressions of virtual reality as an educational tool. The session was conducted in a semi-structured format, which allowed students to freely express opinions while directing the discussion towards specific aspects of the training. Their recommendations were thematically analyzed and integrated into the interpretation of the results and the formulation of training improvements.

### 2.3. Evaluation Procedure

The survey contains 25 questions with multiple-choice answers, which are of the following types: injection skills, technological and digital skills, working with catheters, assessing and managing pain, obstetric skills, hygiene and non-invasive care, and digital motivation and attitudes.

The survey questions are presented in Table 1.

The evaluation instrument used in this study was based on a 5-point Likert-type scale applied to measure self-perceived clinical competence among nursing and midwifery students. In the study [34], the authors developed and validated a clinical competency questionnaire (CCQ) for self-assessment of prospective nursing undergraduates. The instrument uses a Likert scale, allowing students to rate their competencies in various clinical areas.

The survey (Table 1) included a set of statements regarding key clinical skills and tasks commonly performed in healthcare settings. Participants were asked to indicate their level of perceived competence for each task or item by selecting one of the five response options: “Not competent”, “Slightly competent”, “Moderately competent”, “Competent”, or “Very competent”.

To enable quantitative analysis, this five-point verbal scale was transformed into a numerical ordinal scale, a commonly used method in educational and health sciences research. Each response was assigned a score from 0 to 4, representing increasing levels of perceived competence:

- “Not competent” = 0;
- “Slightly competent” = 1;
- “Moderately competent” = 2;
- “Competent” = 3;
- “Very competent” = 4.

This transformation allowed the computation of means, standard deviations, and paired comparisons using parametric tests (e.g., t-test for dependent samples). The approach has been validated and used in prior studies [34,35] involving healthcare students and simulation-based education.

A survey was conducted in two stages: once before the digital and simulation training intervention in the studied specialty (pre-test) and again immediately after its completion (post-test). The pre-test assessed the students’ initial self-assessment of their self-perceived clinical skills, motivation toward digital learning, and openness to using VR and immersive technologies in education. The post-test used the same instrument to capture all changes in perceptions after the intervention. This structure allowed for a direct comparison of the responses of the students from the two groups (experimental group—EG and control group—CG) and an assessment of the perceived impact of blended learning on the development of clinical skills. Responses were analyzed to assess changes in perceived competence, engagement, and attitudes toward immersive learning.

The 25-item self-assessment scale used in this study was developed by the authors to measure perceived clinical competence in key areas of nursing and midwifery education. The content validity of the instrument was ensured through peer review by three faculty members specialized in clinical nursing and midwifery education, who confirmed that the items adequately covered the core clinical tasks and digital readiness competencies related to the curriculum. Construct validity is supported by the clear alignment of the scale items with established theoretical domains such as injection techniques, catheter care, pain management, obstetric skills, hygiene procedures, and digital motivation. The internal consistency of the instrument, measured using Cronbach’s alpha, was α = 0.921 for the Q1–Q22 competence scale and α = 0.943 for the Q23–Q25 technology attitudes scale, indicating the high internal consistency of the instrument. Test–retest reliability analysis was not feasible due to the study design, which included an educational intervention between the two measurements. Furthermore, the short interval (five weeks) between pre- and post-test does not allow for assessment of long-term retention, which is recognized as a limitation.

Welch’s t-test (a modification of Student’s *t*-test) was used for statistical analysis. This test is used when comparing means between two groups of unequal size, which does not assume equal variances (unlike the standard Student’s *t*-test), and is suitable when comparing continuous variables such as mean score, time, or score. In the present study, data analyses were performed using IBM SPSS Statistics, version 26. Welch’s *t*-test was chosen to assess differences between the control and experimental groups, due to the reported inequality of variances of the parameters and different numbers of participants in the study groups.

## 3. Results

### 3.1. Comparison of the Two Groups Before Training and After Training

A Welch’s t-test was conducted to compare the pre-training scores between the control group (*n* = 190) and the experimental group (*n* = 38), due to the unequal sample sizes and potential heterogeneity of variances. As shown in Table 2, no statistically significant differences were observed between the two groups prior to the training intervention across all 25 questionnaire items (*p* > 0.05), confirming baseline equivalence. Following the intervention, statistically significant differences were observed in the majority of items—84% of the questionnaire statements demonstrated significant improvement (*p* < 0.05) in the experimental group compared to the control group, indicating the strong effect of the blended learning approach on students’ self-perceived clinical competence. For Q11, Q12, Q13, and Q14, *p*-value > 0.05, which means that the educational intervention conducted with traditional training alone did not achieve a statistically significant improvement in the students’ self-assessment on these questions.

### 3.2. Analysis of Results by Question Category

#### 3.2.1. Injection Skills (Q1–Q7)

**Figure 6:**
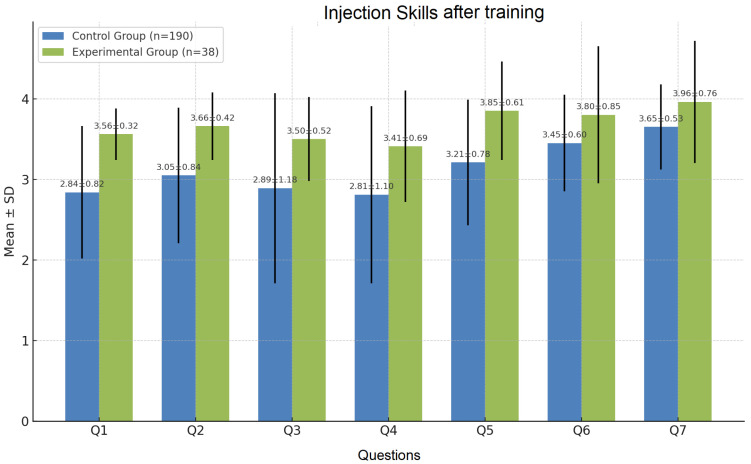
Results obtained after training: basic injection and antiseptic techniques.

These questions address fundamental clinical skills related to injection techniques and antiseptic procedures (Figure 6). All questions show statistically significant improvement in the experimental group (*p* < 0.05), with most reaching *p* < 0.0001. This confirms the effectiveness of the digital module in strengthening injection skills and underscores the benefit of preparatory digital instruction.

#### 3.2.2. Technological and Digital Competencies (Q8)

**Figure 7:**
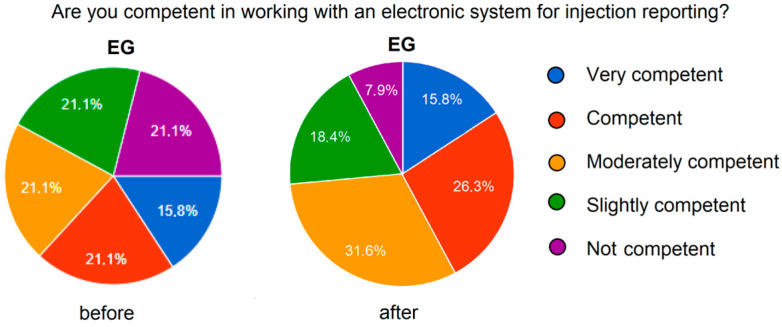
Improvement in self-assessment for working with the electronic system.

Improved self-assessment was also observed in the EG regarding the use of the electronic reporting system following the training (1.89 vs. 2.59, *p* < 0.01). Although the average score remained below three, a statistically significant post-training improvement was evident among students who participated in the flipped classroom intervention (Figure 7).

#### 3.2.3. Catheterization, Pain Assessment, and Pain Management (Q9–Q12)

**Figure 8:**
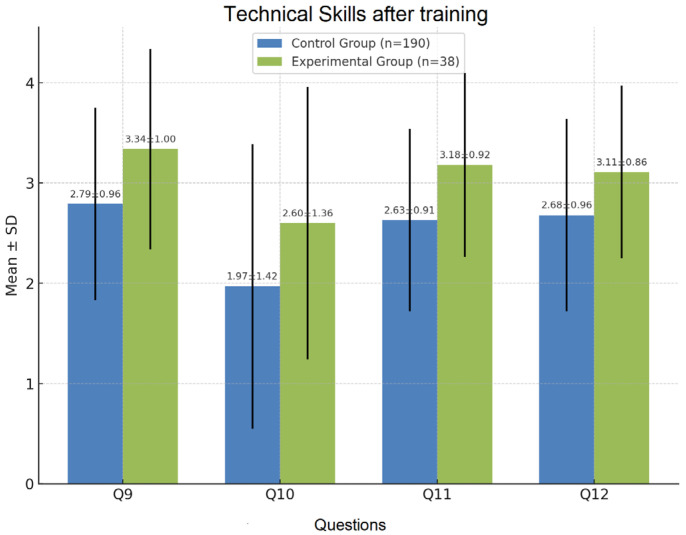
Self-assessment for catheter work, pain assessment, and pain management.

Self-assessment scores for Q9, Q11, and Q12 showed statistically significant improvements in the EG compared to the CG (Figure 8). The results for catheter insertion revealed a statistically non-significant difference (NS), which may be attributed to insufficient hands-on demonstration during the training. This finding highlights the potential need for more targeted and practical instruction in this area of clinical skills.

#### 3.2.4. Midwifery Skills (Q13–Q16)

**Figure 9:**
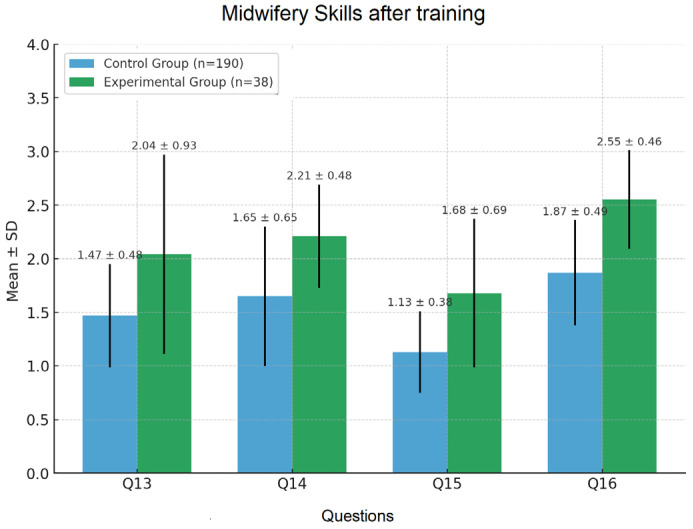
Self-assessment of midwifery skills.

The experimental group showed significant improvements in Q14–Q16 (*p* < 0.05), with Q16 reaching *p* < 0.001 (Figure 9). No significant difference was found for Q13 (uterine palpation), reflecting the complexity of mastering palpation techniques.

#### 3.2.5. Hygiene and Non-Invasive Care (Q17–Q19)

All items in this section demonstrated significant improvements in EG compared to CG (*p* < 0.001), particularly in self-assessments related to dry bed bathing (mean score 3.00 vs. 3.60) and eye/nose/ear hygiene (mean score 3.11 vs. 3.73). These results highlight the effectiveness of the innovative digital materials used to demonstrate daily nursing care procedures.

#### 3.2.6. Digital Motivation and Attitudes (Q20–Q25)

**Figure 10:**
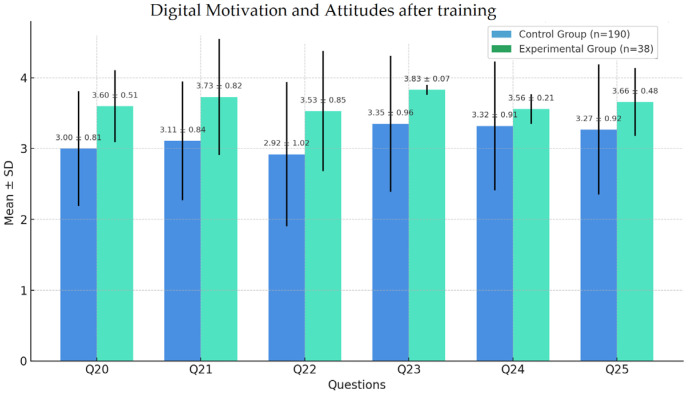
Self-assessment of digital motivation and attitudes.

Significant differences in self-assessment were observed for Q20, Q23, Q24, and Q25 (*p* < 0.001), indicating a stronger digital motivation and more positive attitudes in the experimental group. Q21 and Q22 also reflected higher scores for EG, although the differences were not statistically significant (Figure 10).

**Figure 11:**
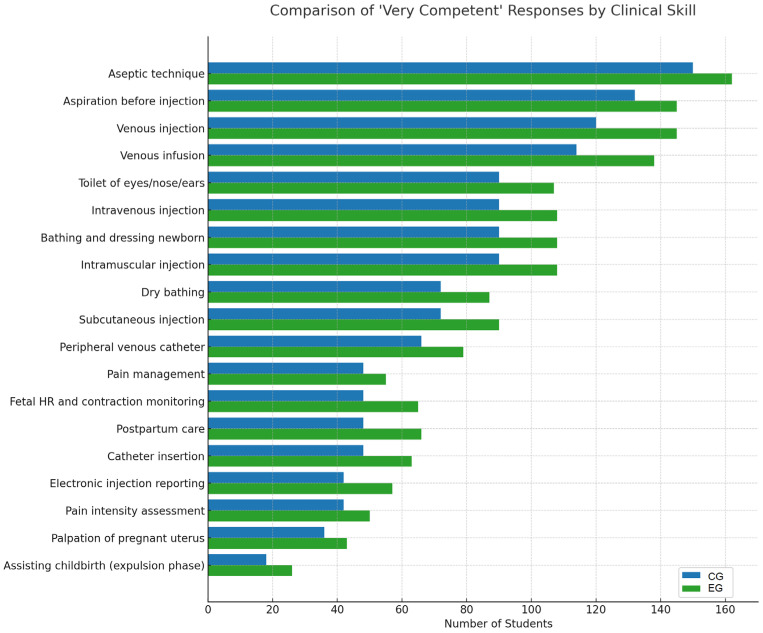
Summary graph of competency assessment questions for both groups.

A comparative chart illustrating the number of students in the EG versus the CG who rated themselves as “highly competent” after the training is presented in Figure 11. The green bars represent the increased confidence of EG students compared to the blue bars of CG students following the educational intervention.

**Figure 12:**
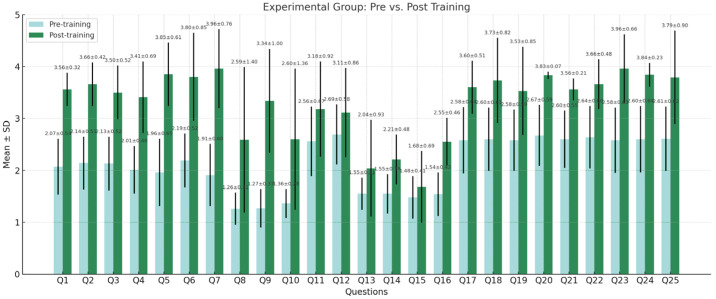
Self-assessment of participants from the experimental group before and after the training.

The graphical results presented in Figure 12 show a significant increase in the number of students from the EG who rated themselves as “very competent” after undergoing training that incorporated video algorithms and an introduction to VR. Most items, particularly those related to technical and digital skills (Q1–Q8, Q20–Q25), demonstrated an average score increase of more than 0.5 points. Statistically significant differences were observed in nearly all measures (*p* < 0.05), highlighting the effectiveness of the flipped classroom approach. The most notable improvements were found in competencies such as venous infusion (Q5), adherence to aseptic technique (Q7), use of electronic reporting systems (Q8), and motivation to use digital tools (Q25).

**Figure 13:**
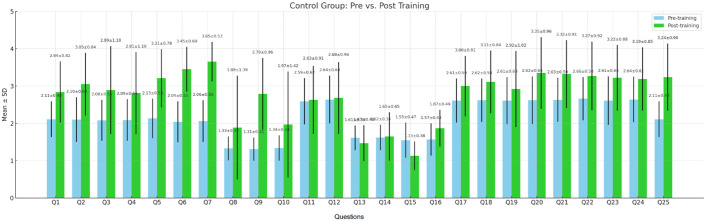
Self-assessment of participants from the control group before and after the training.

The control group (Figure 13), which received training solely through traditional methods such as lectures and anatomical models, also showed improvements in self-assessment following the training; however, these gains were comparatively modest. In many of the assessed items, the increase in average score was below 0.4 and did not reach statistical significance. This highlights the advantages of innovative technologies in fostering the development of complex clinical and digital skills within contemporary healthcare practice. The comparison between groups confirms that the integration of the flipped classroom methodology, visual algorithms, and interactive technologies provides a more effective learning environment and enhances students’ confidence in performing professional tasks.

**Figure 14:**
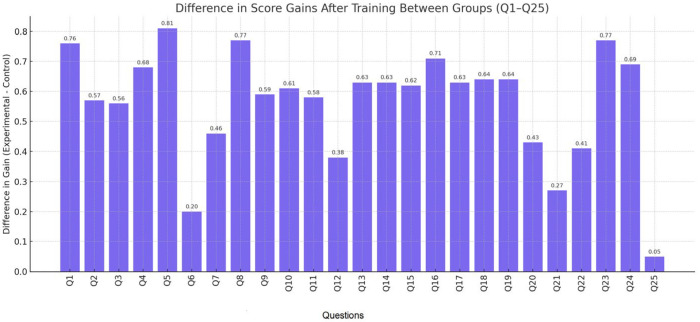
Graph of the difference in grade gain between the experimental and control groups.

Figure 14 presents a graph illustrating the difference in gain scores between the experimental and control groups for each question (Q1–Q25). The *Y*-axis represents the magnitude of improvement in the experimental group relative to the control group. This visualization clearly highlights the questions with the most notable differences in favor of the experimental group, such as Q5, Q8, and Q23. For instance, the minimal difference observed in Q25 suggests that both groups achieved similar outcomes for that specific item.

Based on the focus group discussion conducted after the educational intervention, several key recommendations were identified.

The focus group participants were asked the following questions:

What is your view on the effectiveness of simulation training?

How do you perceive the importance of practical training in healthcare education?

How do you assess the role of digital resources in simulations, video algorithms, and online materials (in the flipped classroom)?

How does prior training affect your motivation and confidence?

What do you think is the most effective training format?

Share your thoughts on the educational process in your specialty.

Participants emphasized the need for the following:

- More engaging hands-on practice using realistic simulators, especially for obstetric scenarios;
- Better integration of theoretical content with clinical simulations;
- Access to innovative technologies along with guidance before simulation sessions;
- Small-group feedback sessions following each simulation to reflect on performance and reduce anxiety associated with handling practical tasks.

Participants shared the following reflections:

*“What needs to be practiced in person simply cannot be effectively demonstrated or internalized online. For me, hands-on practice is essential.”*

*“I appreciated the opportunity to prepare in advance with online materials, videos, and presentations. Combined with classroom exercises and real hospital practice, it worked very well.”*

*“The preliminary preparation increased my interest and motivation to learn the procedures. I felt calmer and more confident when performing them later.”*

*“In my opinion, the best format for our training would be in-person practice sessions and online delivery for lectures.”*

## 4. Discussion

### 4.1. Improvement in Clinical Skills and Self-Assessment

The conducted study demonstrates a significant improvement in students’ self-assessment of clinical skills following an educational intervention that incorporated the opportunity for prior familiarization with the learning material through the use of innovative technologies. This effect is particularly notable in the areas of injection techniques (Q1–Q7), technical skills (Q9–Q12), and motivation toward digital tools (Q20–Q25). These findings align with numerous international studies that highlight the benefits of the flipped classroom, virtual reality [36], and simulation-based learning [22,37,38,39,40] in nursing education.

However, it should be noted that the present study utilized medium-fidelity tools (manikins), which may account for the more moderate improvement observed in complex skills such as catheter insertion (Q10) and pain management (Q11).

Despite measurable gains in obstetric competencies—such as fetal monitoring and postpartum care—the scores remained lower than in other domains, indicating reduced student confidence. This highlights the need for more intensive and targeted training.

The results suggest that students are generally receptive to the use of VR and digital platforms in nursing education. However, the lack of a significant difference in certain attitude-related questions may indicate that some students have not yet fully formed their opinions regarding future adoption. This may be due to limited exposure and insufficient information on the potential of VR technologies in clinical education.

### 4.2. Findings Based on Pre-Intervention Assessments in the EG

1. Highly rated skills: the highest frequency of “very competent” responses was seen for the aseptic technique, indicating a strong emphasis on safety during injection procedures. Skills such as aspiration before injection, venous injection, and venous infusion were also rated highly, likely reflecting frequent practice with anatomical models or real-life scenarios.
2. Moderately rated skills: competencies such as intravenous injection, hygiene care (e.g., eye/nose/ear toilet), newborn bathing and dressing, intramuscular injection, dry bathing, and subcutaneous injection received moderate “very competent” ratings. This suggests satisfactory confidence, though possibly insufficient hands-on experience during simulation or clinical placements.
3. Lowest rated skills: assisting childbirth, palpation of the pregnant uterus, pain intensity assessment, electronic reporting, and catheter insertion were among the least confidently rated. This indicates limited exposure to these procedures, lower confidence in performing more specialized or technology-integrated tasks, and possibly a lack of simulated or virtual scenarios or insufficient video-based learning materials.

Overall, the results suggest a dominance in core injection-related skills, which are traditionally included in foundational nursing and midwifery training, but low self-assessment in more specialized areas such as obstetrics, electronic documentation, and pain communication, highlighting the need for targeted interventions through additional practice and simulation.

### 4.3. Findings Based on Post-Intervention Assessments in the EG

1. There was a notable increase in confidence across all competencies among students in the EG compared to the CG. This supports the effectiveness of the flipped classroom approach and confirms the relevance and methodological quality of the video-based materials used in the training modules.
2. The greatest improvements in the EG relative to the CG were observed in aseptic technique, aspiration before injection, eye/nose/ear hygiene, and intramuscular injection. Skills that remained relatively lower even post-training, though still improved, included palpation of the pregnant uterus and assisting childbirth during the expulsion phase.

Unlike prior studies focusing on single methods, our intervention combined asynchronous digital content, VR-based clinical scenarios, and discussion. This blended model enhanced both technical skills and motivation to engage with digital tools (Q23–Q25), confirming its relevance to modern nursing education. While most research evaluates VR and flipped models separately [41], our integrated design underscores the pedagogical synergy of digital methods and peer interaction.

In contrast to the control group, which showed limited or no significant improvements in multiple items (e.g., Q14, Q15, Q16), the experimental group demonstrated widespread competency gains, including in complex clinical reasoning tasks like fetal heart monitoring and childbirth assistance. These outcomes highlight the potential of structured digital interventions to foster both technical and cognitive skills, even in traditionally hands-on domains.

Our contribution is context-specific: a blended learning model tailored for Eastern European nursing education, which often lacks validated scalable frameworks. The use of a domain-specific questionnaire and skill-level analysis—rather than general competence—adds methodological value.

Finally, the focus group feedback highlighted the irreplaceable role of face-to-face learning. Students emphasized hands-on practice with manikins, real clinical settings, and direct interaction as critical to building confidence. This underscores the need to balance innovation with experiential learning.

#### 4.3.1. Development of Clinical Reasoning and Judgment

Structured debriefing and complex scenarios are key to developing critical thinking in nursing education [42]. While our model included instructor feedback, it lacked a formal reflective element, which may explain the modest gains in clinical judgment (Q13–Q16). Future versions could incorporate branching virtual cases and structured debriefing to boost cognitive engagement.

#### 4.3.2. Communication and Teamwork

Although the study focused on individual competence, the increased confidence in nursing care suggests indirect improvements in teamwork and communication, consistent with [41]. However, the absence of role-play activities and interdisciplinary scenarios limits the generalizability of findings related to team-based performance.

#### 4.3.3. Motivation and Digital Readiness

The obtained results show increased motivation and digital readiness (Q23—3.96 ± 0.66), which is in accordance with [20].

#### 4.3.4. Limited Confidence in Midwifery Skills

Although scores for Q13–Q16 improved significantly, confidence in midwifery skills remained low. This is consistent with [42], which highlighted the need for high-quality simulations and purposeful scenarios. The limited practice time and lack of realism may explain the modest results; VR modules with realistic feedback could help to bridge this gap.

Based on the findings of this study and supporting evidence from the literature, the following recommendations can be made:

- Introduce specialized obstetric simulators, including those for simulating childbirth, postpartum care, and critical scenarios;
- Utilize video-based resources and VR scenarios to demonstrate obstetric procedures in a controlled learning environment;
- Organize clinical placements in real maternity wards, providing opportunities for active observation and assisted participation;
- Implement small-group training sessions with structured debriefing after each simulation, focusing on both technical skills and communication competencies.

Participants in the focus group noted that VR simulations could be especially effective for training in injection techniques, particularly intravenous and intramuscular injections, where precision and procedural consistency are critically important. They recommended incorporating virtual scenarios with interactive feedback to simulate various clinical conditions and patients with diverse anatomical features.

### 4.4. Limitations of the Study

The self-assessment tool used relies on the subjective judgments of the participants, which may introduce biases related to socially desirable behavior or overestimation/underestimation of one’s own skills.

The distribution of students by courses (II, III, and IV) does not allow for a completely homogeneous sample, as training in some skills (e.g., catheterization or obstetric care) may have already been completed by some of the respondents, but not by others. This creates different baseline levels of competence, which could affect the results, regardless of the intervention.

The size of the experimental group (*n* = 38), on which the flipped classroom and VR training were applied, is relatively small compared to the control group, which limits the possibilities for generalization.

Due to the structure of the intervention, a formal test–retest reliability assessment could not be conducted, as the same group was exposed to training activities between the two applications of the instrument.

## 5. Conclusions

This study investigates the impact of simulation-based and digital training on the self-assessed clinical competencies of nursing and midwifery students in Bulgaria. The results demonstrate statistically significant improvements in fundamental clinical procedures—particularly injection techniques, aseptic practice, and basic patient care—as well as an increase in students’ motivation and willingness to engage with digital tools and platforms.

The findings highlight the growing importance of digital transformation in healthcare education, even in contexts with limited access to high-fidelity simulation or immersive VR. Students expressed strong interest in adopting immersive learning environments in the future. This underscores the need to expand access to interactive VR simulations, especially for skill areas such as pain management, catheterization, and obstetric care.

A comparative analysis with the results of international research further supports the value of blended simulation and digital methodologies in enhancing clinical confidence, critical thinking, and professional readiness. However, our findings also point to areas requiring further development, including team-based communication training, interactive debriefing, and real-time performance evaluation systems.

Simulation-based training has a positive impact on learners’ self-assessment of skills and motivation. To fully harness the potential of immersive digital tools in nursing and midwifery education, investments in technological infrastructure, localized content, and faculty development will be essential. Future research should focus on long-term skill retention, objective performance assessment, and the integration of high-quality virtual reality into standard curricula.

The findings suggest that flipped classroom models using digital resources can be feasibly implemented in resource-limited settings, providing a foundation for future investment in immersive learning.

## Figures and Tables

**Table 1:** Survey questions.

No.	Question *
Q1	Are you competent in administering a subcutaneous injection?
Q2	Are you competent in administering an intramuscular injection?
Q3	Are you competent in administering a venous injection?
Q4	Are you competent in administering an intravenous injection?
Q5	Are you competent in performing a venous infusion?
Q6	Are you competent in performing aspiration before injection?
Q7	Are you competent in maintaining aseptic technique during injections?
Q8	Are you competent in working with an electronic system for injection reporting?
Q9	Are you competent in placing a peripheral venous catheter?
Q10	Are you competent in inserting a urinary catheter?
Q11	Are you competent in assessing pain intensity?
Q12	Are you competent in managing patient pain?
Q13	Are you competent in palpating the uterus of a pregnant woman?
Q14	Are you competent in monitoring the fetal heart rate and uterine contractions in pregnant women?
Q15	Are you competent in assisting childbirth during the second stage (expulsion phase)?
Q16	Are you competent in providing postpartum care to new mothers?
Q17	Are you competent in performing hygienic care using the dry bathing method?
Q18	Are you competent in performing special hygiene of the eyes, nasal cavity, and ears of a patient?
Q19	Are you competent in bathing and dressing a newborn baby?
Q20	Are you competent in communicating with patients (e.g., a woman in labor/an injection patient, etc.)?
Q21	Are you competent in teamwork?
Q22	Are you competent in maintaining professional ethics in a clinical setting?
Q23	I feel more motivated when the training includes digital elements (videos, presentations, VR simulations).
Q24	Online courses give me the opportunity to study at a time and pace that is convenient for me.
Q25	I am ready to use digital platforms and online training in the future if necessary.

* Note: Table 1 lists all 25 items included in the self-assessment questionnaire. Questions Q1–Q22 assess perceived clinical competencies across the injection skills, technical, communication, and ethical domains, while Q23–Q25 measure students’ motivation and readiness for digital learning environments.

**Table 2:** Comparison of scores between the control and experimental groups before and after the training intervention.

	Before			After		
	Mean * ± SD (CG)	Mean ± SD (EG)	*p*_Before-Value	Mean ± SD (CG)	Mean ± SD (EG)	*p*_After-Value
Q1	2.11 ± 0.48	2.07 ± 0.54	0.7352	2.84 ± 0.82	3.56 ± 0.32	<0.0001
Q2	2.10 ± 0.60	2.14 ± 0.51	0.6637	3.05 ± 0.84	3.66 ± 0.42	<0.01
Q3	2.08 ± 0.55	2.13 ± 0.52	0.5423	2.89 ± 1.18	3.50 ± 0.52	<0.05
Q4	2.09 ± 0.56	2.01 ± 0.46	0.3507	2.81 ± 1.1	3.41 ± 0.69	<0.0001
Q5	2.13 ± 0.53	1.96 ± 0.65	0.1479	3.21 ± 0.78	3.85 ± 0.61	<0.0001
Q6	2.04 ± 0.55	2.19 ± 0.52	0.1275	3.45 ± 0.6	3.80 ± 0.85	<0.0001
Q7	2.06 ± 0.56	1.91 ± 0.60	0.1734	3.65 ± 0.53	3.96 ± 0.76	<0.0001
Q8	1.33 ± 0.32	1.26 ± 0.31	0.1896	1.89 ± 1.39	2.59 ± 1.40	<0.01
Q9	1.31 ± 0.31	1.27 ± 0.37	0.5339	2.79 ± 0.96	3.34 ± 1.00	<0.0001
Q10	1.34 ± 0.34	1.36 ± 0.28	0.7271	1.97 ± 1.42	2.60 ± 1.36	NS **
Q11	2.59 ± 0.62	2.56 ± 0.67	0.8153	2.63 ± 0.91	3.18 ± 0.92	<0.05
Q12	2.64 ± 0.64	2.69 ± 0.58	0.6432	2.68 ± 0.96	3.11 ± 0.86	<0.05
Q13	1.61 ± 0.33	1.55 ± 0.31	0.2861	1.47 ± 0.48	2.04 ± 0.93	NS **
Q14	1.62 ± 0.34	1.55 ± 0.38	0.2969	1.65 ± 0.65	2.21 ± 0.48	<0.01
Q15	1.55 ± 0.47	1.48 ± 0.41	0.3529	1.13 ± 0.38	1.68 ± 0.69	<0.001
Q16	1.57 ± 0.43	1.54 ± 0.42	0.6905	1.87 ± 0.49	2.55 ± 0.46	<0.001
Q17	2.61 ± 0.59	2.58 ± 0.64	0.8164	3.00 ± 0.81	3.60 ± 0.51	<0.001
Q18	2.62 ± 0.58	2.60 ± 0.61	0.8802	3.11 ± 0.84	3.73 ± 0.82	<0.0001
Q19	2.61 ± 0.63	2.58 ± 0.59	0.7905	2.92 ± 1.02	3.53 ± 0.85	<0.001
Q20	2.62 ± 0.64	2.67 ± 0.59	0.6601	3.35 ± 0.96	3.83 ± 0.07	<0.0001
Q21	2.63 ± 0.59	2.60 ± 0.55	0.7478	3.32 ± 0.91	3.56 ± 0.21	NS **
Q22	2.66 ± 0.58	2.64 ± 0.60	0.8766	3.27 ± 0.92	3.66 ± 0.48	NS **
Q23	2.61 ± 0.65	2.58 ± 0.63	0.8267	3.22 ± 0.88	3.96 ± 0.66	<0.0001
Q24	2.64 ± 0.61	2.60 ± 0.64	0.7493	3.19 ± 0.85	3.84 ± 0.23	<0.05
Q25	2.11 ± 0.48	2.61 ± 0.62	0.9618	3.24 ± 0.90	3.79 ± 0.90	<0.001

* Table 2 presents the mean scores (M) and standard deviations (SD) of students’ self-assessed competencies before and after the intervention. Each item was rated on a 5-point Likert scale ranging from 1 (“not competent”) to 5 (“fully competent”). ** NS—not significant.

## Data Availability

The dataset generated during the study is available upon reasonable request.

## Public Involvement Statement

No public involvement in any aspect of this research.

## Guidelines and Standards Statement

This manuscript was prepared in accordance with the Transparent Reporting of Evaluations with Nonrandomized Designs (TREND) statement for non-randomized intervention studies [43].
